# Prevalence of haemoglobin A1c based dysglycaemia among adult community dwellers in selected states in Nigeria: a descriptive cross-sectional study

**DOI:** 10.3389/fendo.2023.1192491

**Published:** 2023-07-20

**Authors:** Ikeoluwapo O. Ajayi, William O. Balogun, Oluwarotimi B. Olopade, Gbadebo O. Ajani, David O. Soyoye, Oladimeji A. Bolarinwa, Michael A. Olamoyegun, Bilqis W. Alatishe-Muhammad, Ifedayo A. Odeniyi, Olukemi Odukoya, Olufemi A. Fasanmade, Funmilayo P. Diyaolu, Erere Otrofanowei, Iorhen Akase, Paul O. Agabi, Adebola Adejimi, Oluwaserimi A. Ajetunmobi, Kabir A. Durowade, Emmanuel O. Gabriel-Alayode, Azeez O. Ibrahim, Okechukwu O. Ezekpo, Toyin O. Elegbede, Ayodeji O. Lamidi, Funmilayo A. Owolabi, Adebimpe O. Yusuf, Tajudin A. Adetunji, Ayodele J. Ogunmodede, Abolore H. Ameen, Abayomi S. Biliaminu, Sanni Nasiru

**Affiliations:** ^1^ College of Medicine, University of Ibadan, Ibadan, Nigeria; ^2^ Department of Medicine, University College Hospital Ibadan, Ibadan, Nigeria; ^3^ College of Medicine, University of Lagos, Lagos, Nigeria; ^4^ College of Medicine and Health Sciences, Afe Babalola University, Ekiti, Nigeria; ^5^ College of Health Sciences, Obafemi Awolowo University, Ile-Ife, Nigeria; ^6^ College of Health Sciences, University of Ilorin, Ilorin, Nigeria; ^7^ Department of Medicine, Ladoke Akintola University of Technology, Ogbomosho, Nigeria; ^8^ Directorate of Planning, Research and Statistics, Kwara State Ministry of Education, Ilorin, Nigeria; ^9^ Department of Medicine, Federal Teaching Hospital Ido-Ekiti, Ido-Ekiti, Nigeria; ^10^ Department of Medicine, University of Ilorin Teaching Hospital, Ilorin, Nigeria

**Keywords:** pre-diabetes, undiagnosed diabetes, prevalence, adult Nigerians, selected states, haemoglobin A1c

## Abstract

**Background:**

Type 2 diabetes mellitus (T2DM) is a disease of public health importance globally with an increasing burden of undiagnosed pre-diabetes and diabetes in low- and middle-income countries, Nigeria in particular. Pre-diabetes and diabetes are established risk factors for cardiovascular complications. However, data are scanty on the current prevalence of these conditions in Nigeria, based on haemoglobin A1c (HbA1c) diagnosis as recommended by the WHO in 2009. We aimed to determine the prevalence of pre-diabetes, diabetes, and undiagnosed diabetes among the adult population of Nigeria using HbA1c.

**Methodology:**

A cross-sectional, multi-site population study was carried out in selected states in Nigeria (namely, Ekiti, Lagos, Osun, Oyo, and Kwara states) involving 2,708 adults (≥18 years) in rural and urban community dwellers, without prior diagnosis of pre-diabetes or diabetes. Participants with ongoing acute or debilitating illnesses were excluded. Data were collected using an interviewer-administered pretested, semi-structured questionnaire. Socio-demographic, clinical (weight, height, blood pressure, etc.), and laboratory characteristics of participants including HbA1c were obtained. Data were analysed using STATA version 16.

**Results:**

The mean age of participants was 48.1 ± 15.8 years, and 65.5% were female. The overall prevalence of pre-diabetes and undiagnosed diabetes was 40.5% and 10.7%, respectively, while the prevalence of high blood pressure was 36.7%. The prevalence of pre-diabetes was the highest in Lagos (48.1%) and the lowest in Ekiti (36.7%), while the prevalence of diabetes was the highest in Kwara (14.2%) and the lowest in Ekiti (10%). There was a significant association between age of the participants (p< 0.001), gender (p = 0.009), educational status (p = 0.008), occupation (p< 0.001), tribe (p = 0.004), marital status (p< 0.001), blood pressure (p< 0.001), and their diabetic or pre-diabetic status. Independent predictors of diabetes and pre-diabetes include excess weight gain, sedentary living, and ageing. Participants within the age group 45–54 years had the highest total prevalence (26.6%) of pre-diabetes and diabetes.

**Conclusion:**

Over half of the respondents had pre-diabetes and diabetes, with a high prevalence of undiagnosed diabetes. A nationwide screening campaign will promote early detection of pre-diabetes and undiagnosed diabetes among adult Nigerians. Health education campaigns could be an effective tool in community settings to improve knowledge of the risk factors for diabetes to reduce the prevalence of dysglycaemia.

## Introduction

The growing burden of diabetes mellitus (DM), particularly type 2 (T2DM), has become a thing of global concern. Approximately 537 million people are affected by DM with a projected increase to 783 million by 2045 (1). Africa is predicted to have the largest percentage rise (134%) in diabetes prevalence from 24 million in 2019 to 55 million in 2045 when compared to other regions of the world (1). This constitutes a great public health and economic concerns, as the African continent harbours many poorly resourced countries.

Impaired fasting glucose (IFG) and impaired glucose tolerance (IGT) are pre-diabetic states, which can exist undetected for many years with irreversible damage to vital organs of the body (2, 3).

Pre-diabetes will progress to overt T2DM in approximately 25% of patients within 3–5 years, and up to 70% of individuals with pre-diabetes will develop overt diabetes within their lifetime in the absence of intervention (2). The current global prevalence of IGT is 10.6%, out of which about three-quarters live in low- and middle-income countries (LMICs), with a projected rise up to 11.4% by 2045 (1). A recent meta-analysis involving 15 studies in Nigeria, based on American Diabetes Association and World Health Organization criteria, reported the pooled prevalence of pre-diabetes as 13.2% (CI: 5.6%–23.2%) and 10.4% (CI: 4.3%–12.9%), respectively (4). This translates to 15.8 million and 12.5 million adult Nigerians living with pre-diabetes. Notably, there is a wide heterogeneity among the included studies, and only two of them used haemoglobin A1c (HbA1c) for the diagnosis of pre-diabetes. A study performed in Ibadan, southwest zone, on the prevalence and predictors of pre-diabetes among the administrative staff of a tertiary health centre reported a prevalence of 22.3%, suggesting there could be a wide variation depending on the socio-economic group studied (5). In that study, male sex, positive family history of diabetes mellitus, alcohol intake, and inadequate moderate-intensity physical activity were identified in the study as independent predictors.

Pre-diabetes (IGT and IFG) indicate an already heightened risk of cardiovascular disease (3, 6, 7), and their recognition compels urgent interventions that can lead to the prevention of type 2 diabetes and its complications (8). Fasting blood glucose and 2-h oral glucose tolerance test (OGTT) are the traditional methods of screening and diagnosis of diabetes. Following the recommendation by an expert committee in 2009, the use of HbA1c for the diagnosis of diabetes and pre-diabetes was also approved for the diagnosis of these dysglycaemic states (9). Also, potential limitations due to haemoglobinopathy have been minimised with newer standardised methods and assays (10).

## Methods

**Figure 1:**
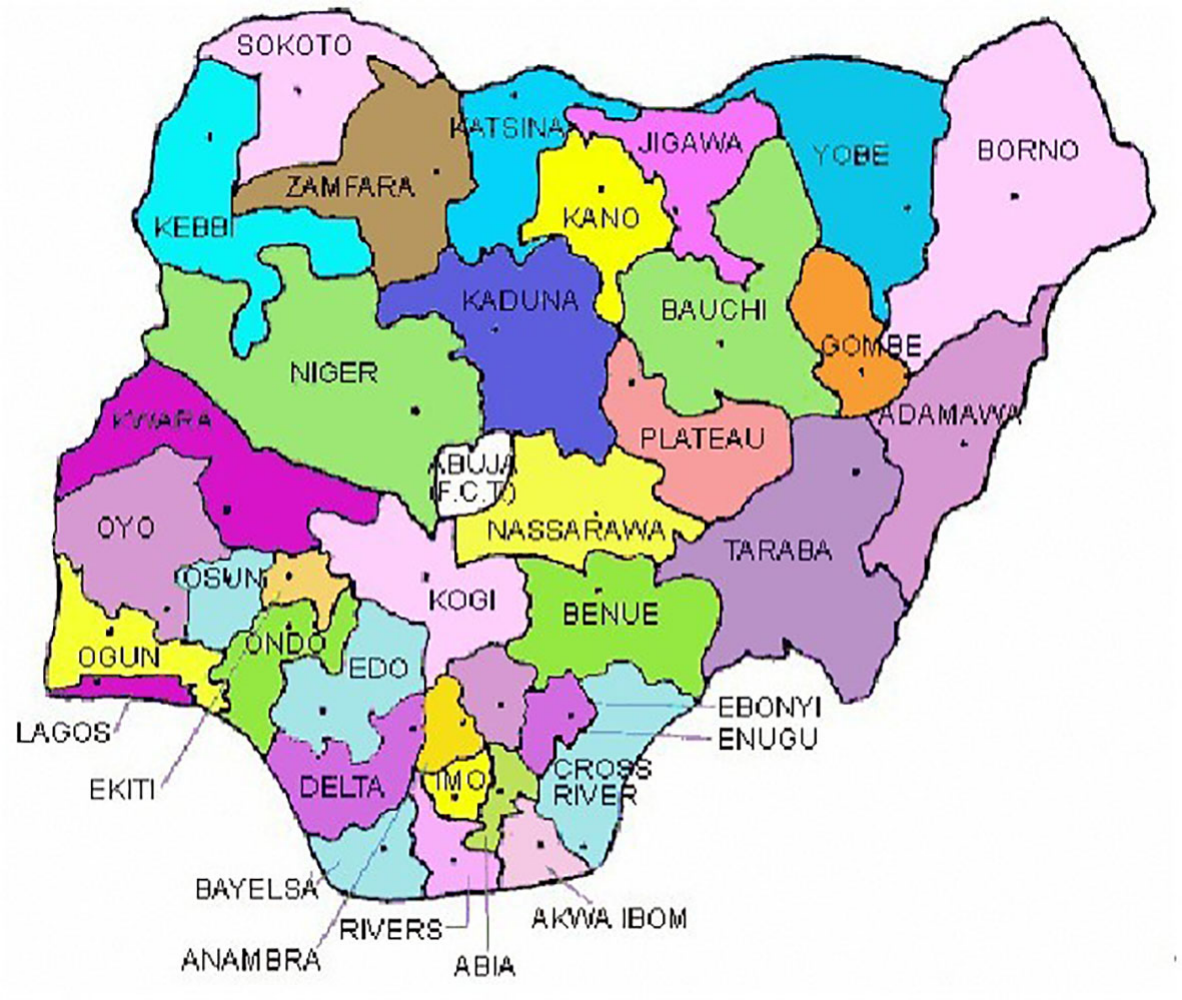
Map of Nigeria showing the data collection sites.

The use of HbA1c as a means of diagnosis of dysglycaemia by clinicians is gradually increasing in Africa including Nigeria, but sparse data exist in our region about HbA1c-based prevalence of pre-diabetes and diabetes. Additionally, no large-scale A1c-based regional or national study exists to date on the burden of pre-diabetes and undiagnosed diabetes among Nigerians. Pre-diabetes is reversible with appropriate lifestyle interventions and selected pharmacology and thus can potentially reduce the burden of diabetes and its complications. To this end, we undertook a multistate large-scale cross-sectional study involving the adult population in southwestern and northcentral Nigeria, and here, we present the findings (**Figure 1**).

### Study design

The study was a population-based descriptive cross-sectional study.

### Study population

The study population included individuals aged ≥18 years and residents in the selected states without a prior diagnosis of pre-diabetes or diabetes mellitus. They had no ongoing acute or chronically debilitating illness.

### Sample size and sampling procedure

Previous studies suggest that the prevalence of pre-diabetes in Nigeria ranged between 2.0% and 37.2%, while that of diabetes varied from 5% to 10%. ^8^ For a conservative sample size, we assumed the upper limits of the prevalence. Therefore, we assumed that the prevalence of the glycaemic disorder is 37.2%, at a 95% confidence level, 2.5% error margin, and a design effect of 1.5.


n=(Z_(α)^2(p*q))/d^2*D,

where Zα is the standard normal deviate at 95% confidence level = 1.96, P is the prevalence of pre-diabetes or diabetes (glycaemic disorder) = 37.2% = 0.372, q = 1 − p, and d is the degree of precision = 2.5% = 0.025.


n=(〖1.96〗_ ^2(0.372*0.628))/〖0.025〗^2*1.5

n=2,151

A minimum sample size of 2,151 was obtained.

A non-response rate of 20% was estimated to give a sample size of 2,151/((1 − 0.2)) = 2,689.

### Sampling technique

A multistage sampling technique was used to select communities from all parts of each selected state to represent both the urban and rural settlements of the states. The states were purposively selected based on the availability of researchers and willingness to participate in the study.

Stage 1: each state was to select a minimum of two local government areas (LGA) by simple random sampling.

Stage 2: the selected LGAs were divided into rural and urban LGAs, and a minimum of 300 participants were assigned to each state.

Stage 3: a sampling frame listing all political wards in the selected LGAs was developed. Two wards were selected using a simple random sampling technique.

### Instrument

A pretested, semi-structured questionnaire was used to obtain the data. Data were collected electronically using Android phones loaded with the questionnaires and captured on REDCAP software version 5.19.7. The questionnaire was developed using information from literature, researchers’ knowledge of the topic, and adaptation of questions from past related studies. The draft questionnaire was face validated by experts to ensure the adequacy of the questions to provide answers to the study objectives. It was then pretested in a community that was excluded from the study sites to ensure the clarity, comprehensiveness, conciseness, and consistency of questions. The questionnaire was translated into the local language and back-translated into English to ascertain the accuracy of the translation. Information collected from the study participants included socio-demographic characteristics (age, gender, etc.), clinical (weight, height, blood pressure, etc.), and laboratory profiles of the individuals (glycated haemoglobin). Prior history of gestational diabetes, hypertension, family history of diabetes, dietary and lifestyle history, and selected disease screening practices including diabetes were collected.

### Method of data collection

Mobilisation campaigns were performed in the communities through walk-round sensitisation in the selected wards. Adult residents were invited to the primary health care centre nearest to them for interview and screening after written informed consent was obtained from each eligible individual. The interviewers were graduates of tertiary institutions and were well-versed in community-based quantitative data collection. They were trained to take anthropometric measurements: weight, height, waist circumference, hip circumference, and blood pressure. All interviewers were fluent in English and Yoruba languages. Weights were measured to the nearest 0.1 kg with portable bathroom scales (HARSON), which were validated with an object with a known weight.

The scales were also regularly checked and adjusted to zero after every 10th measurement. The participants were weighed wearing light clothing, with emptied pockets, no attachment to their clothing, and without shoes. Heights were measured with a mobile stadiometer placed on a level ground against a flat vertical surface, with participants standing erect without shoes and with the eyes looking horizontally and the feet together on a horizontal level. Heights were recorded to the nearest 0.1 cm. Standardisation checks on the height boards were performed periodically during the study period. Body mass index (BMI) was calculated using the following formula: BMI = weight/(height in meters)^2^ (kg/m^2^). BMI was classified as normal, 18.5–24.9 kg/m^2^; overweight, 25–29.9 kg/m^2^; class I obesity, 30–34.9 kg/m^2^; class II obesity, 35–39.9 kg/m^2^; class III obesity, 40 kg/m^2^ or higher.

Waist circumference was measured using a flexible tape measure, with the subject standing and breathing normally. The examiner stood on the right side of the subject, and then the inferior margin of the last rib and iliac crest were located and identified with a pen. A tape measure was placed at the midpoint between the landmarks in a way that the tape was in a horizontal plane parallel to the floor. The waist circumference was measured and read at the level of the tape to the nearest 0.1 cm. A waist circumference greater than or equal to 94 cm in men and greater than or equal to 80 cm in women was taken as indicative of truncal obesity.

Hip measurement was made while the participant was in a relaxed standing position with the feet together. The measuring tape was placed horizontally around the maximum circumference of the buttocks. The hip circumference was measured and read at this level to the nearest 0.1 cm.

Systolic and diastolic blood pressure were assessed using an automated digital blood pressure monitor (OMRON M3) with an appropriately sized cuff for participants’ arms. The measurements were taken after each participant had rested for at least 5 min, were well seated, resting their back with feet on the ground, and not distracted. Blood pressure was measured twice using the left arm, with an interval of 2 min. Blood pressure (BP) measured was categorised using standard guidelines such as The Seventh Report of the Joint National Committee on Prevention, Detection, Evaluation, and Treatment of High Blood Pressure: the JNC 7 report. In this study, hypertension was defined as an average of two measurements of systolic and/or diastolic blood pressure (second and third blood pressure measurement) that is ≥140/90 mmHg in any adult or self-reported treatment of hypertension with antihypertensive medication taken in the past weeks. Phlebotomists were recruited in each state to collect 2 mL of venous blood under an aseptic condition from the participants out of which drops were used to determine their random blood glucose (RBG). The remaining blood samples were stored in EDTA bottles, which were placed in cold boxes and transported to a central laboratory for the estimation of HbA1c.

Glycated haemoglobin was determined using a mobile point-of-care equipment (DCA Vantage Analyzer by Siemens, Erlangen, Germany) certified by the National Glycohemoglobin Standardization Program (NGSP) and International Federation of Clinical Chemistry and Laboratory Medicine (IFCC). In diabetes, the glucose moiety is gradiently added to the haemoglobin chain of the red blood cells, and this can be retrospectively assessed using the HbA1c, reflecting blood glucose level corresponding to the lifespan of a red blood cell. Siemens’ DCA HbA1c test is based on an immunoassay technique and is standardised to both NGSP and IFCC reference methods. HbA1c tests using the device demonstrated tight precision, with CVs of 2.0% or less in the important clinical ranges for diagnosing and monitoring. These results exceed the acceptance criteria for precision as specified in the Clinical and Laboratory Standards Institute (CLSI) guidelines. They also demonstrated excellent correlation (R = 0.99) of the DCA HbA1c test with the NGSP-certified Tosoh G7 central laboratory HbA1c method. Although potential limitation due to haemoglobinopathy has been minimised with newer standardised methods and assays (9), the possibility of error in those with haemoglobinopathy cannot be completely eliminated (1). In addition, participants had RBG levels estimated using a glucometer (Accu-Chek Active glucometer and strips). An RBG level of 200 mg/dL or higher indicates diabetes.

Phlebotomists were recruited in each state to collect 2 mL of venous blood under an aseptic condition from the participants out of which drops were used to determine their RBG. The remaining blood samples were stored in EDTA bottles, which were placed in cold boxes and transported to the laboratory for the estimation of HbA1c. Pre-diabetes is defined with glycated haemoglobin levels 5.7%–6.4% and undetected diabetes ≥6.5% (1, 3, 11). An RBG level of 200 mg/dL or higher indicate diabetes (1).

### Data analysis

Data were analysed using the STATA version 16 package program. Descriptive statistics such as means and standard deviation were used to summarize categorical variables, while proportions were used for qualitative variables. The associations between the categorical variables were performed using the chi-square test. The comparison of means was tested using Student’s t-test for quantitative variables. Associations between continuous variables were determined using correlation analysis. Multiple logistic regression was used to determine predictors of pre-diabetes and diabetes.

All statistical analyses were carried out at a level of significance<0.05.

### Ethical considerations

Ethical approval was obtained from the Ethical Committees in all the selected states and the National Health Research Ethics Committee of Nigeria (NHREC) [Ref No.: NHREC/01/01/2007]. Permission was obtained from the local heads of the communities and the primary health care unit of the LGA secretariats. Written informed consent was obtained from each participant before commencing with the data collection, and participants had the right to withdraw from the study at any time during data collection.

## Results

**Figure 2:**
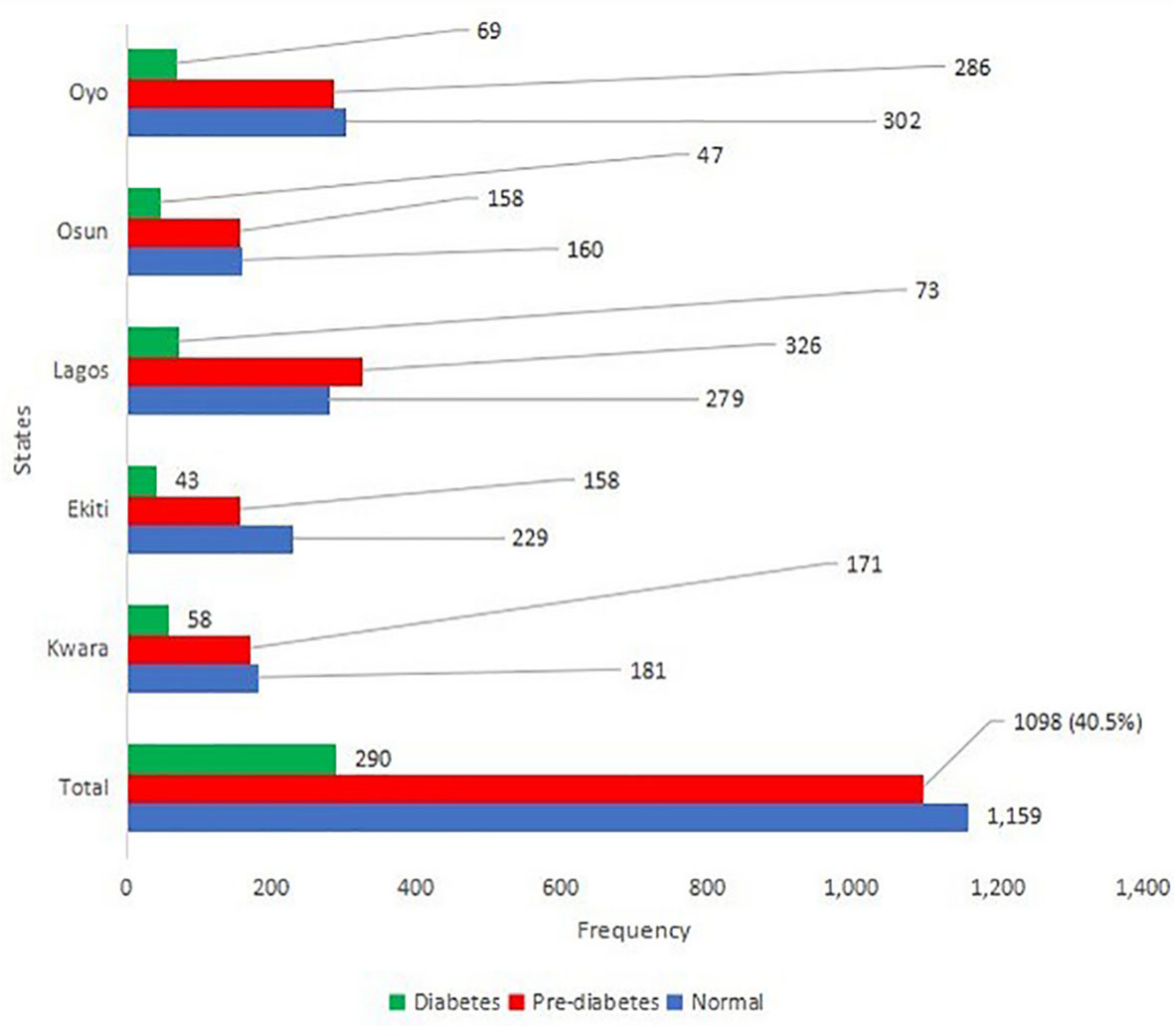
Prevalence of diabetes, pre diabetes and normoglycemia among participants in each state.

A total of 2,708 adults were studied in the selected five states. Out of the 2,708 participants, 1,759 (65.5%) were female, 2,325 (86.6%) were Yoruba, and 1,917 (71.4%) were married. The mean age of the participants was 48.1 ± 15.8 years. A total of 872 (32.5%) participants had up to tertiary education, while 393 (14.7%) never attended school (**Table 1**). Participants who had never smoked were 2,465 (92.3%), while 141 (67.1%) of those that signified to have smoked before were current smokers. A total of 1,994 (74.8%) of the participants had never taken alcohol before, and 209 (53.2%) out of those who consumed alcohol were occasional drinkers. Also, 2,474 (92.7%) of the participants consumed fruits at least once weekly. The overall prevalence of high blood pressure was 36.7%. The prevalence of pre-diabetes was the highest in Lagos (48.1%) and the lowest in Ekiti (36.7%), while the prevalence of diabetes was the highest in Kwara (14.2%) and the lowest in Ekiti (10%) (**Table 2** and **Figure 2**). Out of the 290 participants with diabetes, 244 (84.1%) reaffirmed no prior knowledge of having diabetes, giving a true prevalence of undiagnosed diabetes as 9.6%.

**Table 1:** Frequency distribution of socio-demographic characteristics of participants.

Socio-demographics	Frequency (n) 2,708	Percentage (%)
Age group (years)		
≤24	191	7.1
25–34	366	13.5
35–44	590	21.8
45–54	629	23.2
55–64	468	17.3
65–74	281	10.4
≥75	155	5.7
Missing	28	1.0
Gender		
Male	925	34.2
Female	1,759	65.0
Missing	24	0.8
Educational status		
Never attended	393	14.5
Primary	493	18.2
Secondary	793	29.3
Tertiary	872	32.2
Postgraduate	130	4.8
**Missing**	27	1.0
Occupation		
Civil/public servant	433	16.0
Farming	162	6.0
Business	1,435	53.0
Professional	268	9.9
Student	146	5.4
Retired/unemployed	264	9.7
Tribe		
Yoruba	2,325	85.6
Hausa	32	1.2
Igbo	213	7.9
Others	114	4.3
Missing	24	1.0
Religion		
Christian	1,647	60.8
Islam	1,032	38.1
Others	5	0.2
Missing	24	0.9
Marital status		
Single	395	14.6
Married/cohabiting	1,939	71.6
Divorced/widowed/separated	374	13.8

**Table 2:** Lifestyle and clinical characteristics of participants.

Lifestyle characteristics	Frequency (n) 2,708	Percentage (%)
Smoking history		
**Ever smoked** Yes No Missing	205 2,465 38	7.6 91.0 1.40
**Currently smokes (N = 205)** Yes No	69 136	33.5 66.5
**Regular contact with someone who smokes** Yes No Missing	275 2,394 39	10.2 88.4 1.4
**Ever taken alcohol** Yes No Missing	673 1,994 41	24.9 73.6 1.5
**Alcohol consumption pattern** Daily Weekly Monthly Occasionally Others Missing	N = 673 61 86 30 209 7 287	9.5 12.8 4.5 31.1 1.0 42.6
**Ever taken alcoholic local concoctions for ailments** Yes No Missing	700 1,966 42	25.8 72.6 1.6
**Eats fruit** Yes No Missing	2,475 198 35	91.4 7.3 1.3
**High blood pressure** No Yes Missing	1,625 994 89	60.0 36.7 3.3
**Glycaemic status** Normoglycaemia Pre-diabetes Diabetes Missing	1,159 1,098 290 161	42.8 40.5 10.7 6.0
**BMI** Mean (SD) Underweight n (%) Normal n (%) Overweight n (%) Obese n (%)	25.3 ( ± 5.6) 196 1,186 744 482	7.5 45.5 28.5 18.5

BMI, body mass index.

The ratio of previously diagnosed to newly diagnosed diabetes was 1.11/9.60 = 0.11. The prevalence of pre-diabetes and diabetes was the highest in Lagos State at 29.7% and 25.2%, respectively (**Figure 2**). The study showed statistically significant associations between the age of the participants (p< 0.001), gender (p = 0.009), educational status (p = 0.008), occupation (p< 0.001), tribe (p = 0.004), marital status (p< 0.001), blood pressure status (p< 0.001), and their diabetic or pre-diabetic status. Participants within the age group 45–54 years had the highest total prevalence (26.6%) of pre-diabetes and diabetes, and 765 (56.2%) of participants who were either pre-diabetic or diabetic had high blood pressure. No participant within the age range ≤24 years had diabetes as shown in **Table 3**.

**Table 3:** Prevalence of pre-diabetes and diabetes among participants.

	Pre-diabetes		Diabetes		Total		p-Value	X^2^
Age group (years)	n	%	n	%	n	%	<0.001	85.14
15–24	63	5.7	0	0.0	63	4.5		
25–34	108	9.8	4	1.4	112	8.1		
35–44	234	21.3	31	10.7	265	19.1		
45–54	287	26.1	83	28.6	369	26.6		
55–64	209	19.1	85	29.3	294	21.2		
65–74	113	10.3	62	21.4	175	12.6		
≥75	84	7.7	25	8.6	109	7.9		
Gender							0.009	6.92
Male	365	33.2	73	25.2	438	31.6		
Female	733	66.8	217	74.8	950	68.4		
Educational status							0.008	13.80
Never attended	163	14.9	61	21.0	224	16.2		
Primary	202	18.4	65	22.4	267	19.1		
Secondary	314	28.6	74	25.5	388	28.0		
Tertiary	353	81.2	82	18.8	435	31.3		
Postgraduate	66	89.2	8	10.8	74	5.3		
Occupation							<0.001	27.36
Civil servant	169	15.4	46	15.9	215	15.5		
Farming	50	4.5	10	3.5	60	4.3		
Business	603	54.9	159	54.8	762	54.9		
Professional	118	10.8	21	7.2	139	10.0		
Student	43	3.9	0	0.0	43	3.1		
Retired/unemployed	115	10.5	54	18.6	169	12.2		
Tribe							0.004	13.14
Yoruba	942	85.8	263	90.7	1,205	86.8		
Hausa	3	0.3	3	1.0	6	0.4		
Igbo	98	8.9	21	7.2	119	8.6		
Others	55	5.0	3	1.0	58	4.2		
Religion							0.616	0.97
Christian	666	60.7	170	58.6	836	60.2		
Islam	430	39.2	120	41.4	550	39.6		
Others	2	0.2	0	0.0	2	0.2		
Marital status							<0.001	46.12
Single	141	12.8	4	1.38	145	10.5		
Married/cohabiting	797	72.6	211	72.8	1,008	72.6		
Divorced/widowed/separated	160	14.6	75	75.9	235	16.9		
Ever smoked							0.120	2.42
Yes	96	8.7	17	5.9	112	8.1		
No	1,002	91.3	273	94.1	1.275	91.9		
Currently smokes							0.154	2.03
Yes	29	29.7	2	12.5	29	27.1		
No	67	70.3	15	87.5	78	72.9		
Regular contact with someone who smokes							0.495	0.46
Yes	93	8.5	21	7.2	114	8.2		
No	1,005	91.5	269	92.8	1,273	91.8		
Ever taken alcohol							0.080	3.06
Yes	278	25.3	59	20.3	336	24.3		
No	820	74.7	231	79.7	1,049	75.7		
Ever taken alcoholic local concoctions for ailments							0.208	1.58
Yes	282	25.7	64	22.1	345	24.9		
No	816	74.3	226	77.9	1,040	75.1		
Eats fruits							0.128	2.31
Yes	1,031	93.9	265	91.4	1,293	93.4		
No	67	6.1	25	8.6	92	6.6		
High blood pressure							<0.001	35.20
Yes	662	60.3	118	40.6	765	56.2		
No	436	39.7	172	59.4	596	43.8		

There was a significant association between their age (p< 0.001), gender (p = 0.003), educational status (p = 0.025), occupation (p< 0.001), marital status (p< 0.001), blood pressure status (p< 0.001), and their knowledge of whether they have diabetes or not. Participants within the age range 55–64 years had the highest prevalence of undiagnosed diabetes (30.7%), and 57.1% of the participants with high blood pressure had undiagnosed diabetes (**Table 4**). The multivariable logistic regression analysis is shown in **Table 5**, demonstrating the relationship between predictor variables and diabetes (**Table 5A**) and pre-diabetes (**Table 5B**). Compared to the reference categories, women, those who had secondary or higher education, retired and unemployed, not having high blood pressure, and overweight/obese have higher odds of developing diabetes. For pre-diabetes, the predictor variables were 45, 64, or >75 years of age, married, widowed, obese, retired, and those with occupations such as civil servants, petty trading/business, and professionals.

**Table 4:** Prevalence of undiagnosed diabetes among participants.

	Diagnosed	%	Undiagnosed	%	p-Value	X^2^
	n		n			
**Age group (years)**					0.359	5.49
15–24	46	15.9	244	84.1		
25–34	1	2.12	3	1.2		
35–44	2	4.4	29	11.9		
45–54	15	32.6	68	27.9		
55–64	10	21.7	75	30.7		
65–74	12	26.1	50	20.5		
≥75	6	13.0	19	7.8		
**Gender**					0.559	0.34
Male	10	21.7	63	25.8		
Female	36	78.3	181	74.2		
**Educational status**					0.565	2.96
Never attended	11	23.9	50	20.5		
Primary	13	28.3	52	21.3		
Secondary	10	21.7	64	26.2		
Tertiary	12	26.1	70	28.7		
Postgraduate	0	0.0	8	3.3		
**Occupation**					0.627	2.60
Civil servant	6	13.0	40	16.4		
Farming	3	6.5	7	2.9		
Business	27	58.7	132	54.1		
Professional	2	4.3	19	7.8		
Retired/unemployed	8	17.4	46	18.9		
**Tribe**					0.731	1.29
Yoruba	42	91.3	221	90.6		
Hausa	0	0.0	3	1.2		
Igbo	4	8.7	17	7.0		
Others	0	0.0	3	1.2		
**Religion**					0.022*	5.27
Christian	34	73.9	136	55.7		
Islam	12	26.1	108	44.3		
**Marital status**					0.387	5.24
Single	0	0.0	4	1.6		
Married/cohabiting	30	65.2	181	74.2		
Divorced/widowed/separated	16	34.8	59	24.2		
**Ever smoked**					0.836	0.04
Yes	3	6.5	14	5.7		
No	43	93.5	230	94.3		
**Currently smokes**					0.468	0.53
Yes	0	0.0	2	15.4		
No	3	100	11	84.6		
**Regular contact with someone who smokes**					0.678	0.17
Yes	4	8.7	17	7.0		
No	42	91.3	227	93.0		
**Ever taken alcohol**					0.292	1.11
Yes	12	26.1	47	19.3		
No	34	73.9	197	80.7		
**Ever taken alcohol local concoctions for ailments**					0.404	0.70
Yes	8	17.4	56	23.0		
No	38	82.6	188	77.0		
**Eats fruit**					0.260	1.27
Yes	44	95.7	221	90.6		
No	2	4.3	23	9.4		
**High blood pressure**					0.065	3.41
Yes	33	72.1	133	57.1		
No	13	27.9	105	42.9		

*Significant at p< 0.05.

**Table 5:** Multivariable analysis showing relationship between predictor variables and (a) diabetes and (b) pre-diabetes.

(a) Diabetes	OR	CI (95%)	p-Value
**Age group (years)** ≤24 25–34 35–44 45–54 55–64 65–74 ≥75	**1.00** 0.06 0.28 0.79 1.16 1.49 **1.00**	0.02–0.17 0.16–0.50 0.48–1.28 0.71–1.89 0.89–2.50	<0.001* <0.001* 0.335 0.557 0.129
**Gender** Male Female	**1** 1.61	1.22–2.13	0.001*
**Educational status** Never attended Primary Secondary Tertiary Postgraduate	**1** 0.83 0.55 0.56 0.35	0.57–1.22 0.38–0.79 0.39–0.80 0.16–0.75	0.348 0.001* 0.001* 0.007*
**Occupation** Junior civil/public servant Senior civil/public servant Farming Artisanry Petty trading Business Professional Student Retired Unemployed Apprenticeship	**1** 1.12 0.65 0.71 1.33 1.21 0.78 **1** 2.47 2.28 1.11	0.60–2.07 0.29–1.43 0.38–1.31 0.79–2.23 0.69–2.12 0.41–1.49 1.35–4.54 1.18–4.41 0.31–4.02	0.727 0.285 0.270 0.276 0.503 0.456 0.004* 0.014 0.875
**Marital status** Single Married Divorced Widowed Separated Cohabiting	**1** 11.93 6.08 26.02 25.34 22.81	4.41–32.30 0.64–57.80 9.37–72.22 6.27–102.52 2.06–252.13	<0.001* 0.116 <0.001* <0.001* 0.011
**High blood pressure** Yes No	**1** 2.71	2.11–3.49	<0.001*
**BMI** Underweight Normal Overweight Obese	**1** 1.44 3.26 5.12	0.71–2.93 1.61–6.57 2.55–10.48	0.310 0.001* <0.001*
(b) Pre-diabetes	OR	CI (95%)	p-Value
**Age group (years)** ≤24 25–34 35–44 45–54 55–64 65–74 ≥75	**1.00** 0.87 1.33 1.72 1.67 1.39 2.50	0.60–1.27 0.94–1.88 1.22–2.43 1.17–2.39 0.94–2.06 1.60–3.91	0.482 0.111 0.002* 0.005* 0.097 <0.001*
**Gender** Male Female	**1** 1.07	0.90–1.26	0.451
**Occupation** Junior civil/public servant Senior civil/public servant Farming Artisanry Petty trading Business Professional Student Retired Unemployed Apprenticeship	**1** 1.60 1.03 1.28 1.48 1.87 1.69 0.85 1.78 1.49 1.17	1.07–2.38 0.65–1.63 0.89–1.85 1.05–2.07 1.30–2.69 1.15–2.50 0.53–1.36 1.14–2.77 0.91–2.43 0.50–2.73	0.021* 0.891 0.189 0.024* 0.001* 0.008* 0.497 0.011* 0.110 0.709
**Tribe** Yoruba Hausa Igbo Others	**1** 0.16 1.29 1.51	0.05–0.51 0.97–1.72 1.02–2.24	0.002* 0.085 0.041*
**Marital status** Single Married Divorced Widowed Separated Cohabiting	**1** 1.26 1.26 1.43 0.71 1.08	1.0 –1.58 0.46–3.45 1.05–1.94 0.28–1.76 0.18–6.53	0.049* 0.656 0.022* 0.457 0.935
**BMI** Underweight Normal Overweight Obese	**1** 0.93 1.29 1.55	0.68–1.28 0.93–1.79 1.10–2.19	0.661 0.129 0.013*

BMI, Body mass index.

* Significant at p< 0.05.Bold, Reference variables.

## Discussion

Although it is well established that there is an increasing burden of pre-diabetes and undiagnosed diabetes in sub-Saharan Africa (1), most of the evidence is based on blood glucose as a diagnostic method. This study provides evidence, perhaps for the first time on a large scale, that the prevalence of A1c-based pre-diabetes and diabetes among Nigerians is disturbingly high: 40.5% and 10.7%, respectively. Also, the majority (84.2%) of those with diabetes were previously undiagnosed, implying that the actual prevalence of diabetes was 9.6%. To the best of our knowledge, this is the first study in Nigeria with such large scope to investigate dysglycaemia using HbA1c across states in a region of the country.

The prevalence rate of pre-diabetes in this study is higher than estimates from the International Diabetes Federation (IDF) and those reported from different parts of Africa. Our prevalence rate for diabetes is similar to the finding by Bashir and colleagues in a meta-analysis involving 15 studies in Nigeria (4) and the study by Ekpeyong and co-workers (12). The latest diabetes atlas by the IDF estimated the overall prevalence of pre-diabetes and diabetes in sub-Saharan Africa (SSA) as 17.7% and 4.5%, respectively (1). Studies from different countries in SSA indicate a wide variation in the prevalence of pre-diabetes and diabetes (4, 13–20). Similarly, there are wide variations in the prevalence of pre-diabetes and diabetes reported within Nigeria, ranging between 6.3% and 29% for pre-diabetes and 0.8% and 9.3% for diabetes (4, 5, 21–25). However, these studies used glucose testing methods and not HbA1c. Data on studies based on the latter are very scarce in Nigeria and even the SSA continent. One A1c-based study in Botswana (26) reported a higher prevalence rate of pre-diabetes and diabetes (54.3% and 14.4%, respectively) than our findings.

In Nigeria, a study was performed among 10 communities within a single northern state, mostly Hausa-Fulani ethnic groups, which compared both methods of blood glucose and HbA1c (27). The authors reported an HbA1c-based prevalence of pre-diabetes and diabetes at 9.3% and 3.5%, respectively. The OGTT-based results in the same population were 17.2% and 9.3% for prevalence rates of pre-diabetes and diabetes, respectively. In contrast to most reports, the study by Lawal and co-workers indicate higher prevalence rates of dysglycaemia using blood glucose than A1c (28–31). The reason for the wide disparities between the A1c-based values in that study and our findings could be ethnic variation. Studies have shown that race and ethnicity can influence the results of HbA1c (32–34). Some epidemiological studies among the Northern Hausa-Fulani ethnic groups have reported lower prevalence rates of dysglycaemia compared to ethnic tribes in the southern part of the country (35–39). Adeloye et al. in their meta-analytic work report the prevalence rates in the northern part of Nigeria to range between 2.0% and 4.6%, while that of Southern Nigeria ranges between 3.2% and 8.5% (39). Additionally, the differences could be due to the HbA1c assay methods used in both studies. While we used DCA Vantage analyser based on immunoassays in this study, the work by Lawal was performed using the boronate affinity method. Although none of these methods is the gold standard, both are certified by NGSP and IFCC. However, the A1c result from boronate affinity is based on a ratio of glycated to non-glycated haemoglobin regardless of species and hence could be falsely low in the presence of haemoglobin F (40). Finally, our findings could also reflect the rapid increase in the prevalence of diabetes in the country. Considering the southwest zone where most of the participants in this study resided, a prevalence rate of 0.8% was reported in 1998 from Ibadan (41). However, in 2013, Alebiosu et al. reported a prevalence rate of 5.05% from the southwest zone (42).

The International Expert Committee (IEC) in 2009 recommended a cutpoint of 6.5% for the diagnosis of diabetes and 5.7%–6.4% for pre-diabetes (10). However, this remains controversial due to inconsistent reports of a rise in the incidence of retinopathy at this threshold (43–46). The use of HbA1c and fasting blood glucose has been reported to have limited agreement with OGTT results (47). This limitation and under-performance of HbA1c in African Americans exist even when participants in the study did not have haemoglobinopathy and anaemia (48). Examination of the relationship between HbA1c and plasma glucose concentrations in the USA showed that the African Americans demonstrated a shift of the curve to the right by 0.65%, compared to the non-Hispanic Whites and Mexican Americans, implying a higher threshold of A1c in the African Americans (49, 50). Moreover, the sensitivity of HbA1c to a diagnosis of diabetes is the highest among the Chinese, Asian Indians, and Africans, compared to other races (51–53). The HbA1c-based high prevalence rates of pre-diabetes and diabetes in our study is probably supporting the widely reported high level of HbA1c in Blacks compared to Whites (54), such that with the same cutoff values, more Blacks will be diagnosed with dysglycaemia compared to Whites. As shown by Vega-Vazquez and colleagues, using A1c for diagnosis have more impact on the diagnosis of pre-diabetes than diabetes (31). Our study and the one from Botswana by Omech and co-authors (26) support this suggestion.

The magnitude of undiagnosed diabetes in this study (84.2%) is even higher than the 60% reported by IDF for the African region (1). These participants most likely have type 2 diabetes since that is the form that is insidious. As the prevalence rate of diabetes in Nigeria increases, there is no commensurate improvement in the health system, access to health care, and screening, further worsened by an abysmally low number of people covered by health insurance in the country (55). This is very disturbing considering the consequent risk for cardiovascular disease and other microvascular complications in sub-Saharan Africa (6, 56). When people lack knowledge of their health condition, the consequence is increased mortality and morbidity, which could have been prevented (57, 58). As indicated in our study, older age and hypertensive individuals have been reported as having a higher risk of undiagnosed diabetes in developing countries (59).

Consistent with established knowledge, our study supports that the commonest risk factor for glucose intolerance is excess weight gain (60). Overweight/obesity was common among the predictors for both pre-diabetes and diabetes. The next predictor is physical inactivity indicated in our study as those retired, unemployed, or professionals who require little expenditure of energy. Ageing is also associated with the development of glucose intolerance. The pre-diabetic and diabetic participants in our study were predominantly between 40 and 64 years old. However, age was only an independent predictor in the pre-diabetic group and not diabetes. This could be due to the stronger influence of HbA1c on pre-diabetes (31), underscored by the fact that HbA1c increases with age independent of glucose intolerance (45, 61). Age above 40 years, BMI > 30, and dyslipidaemia have been identified as predictors of pre-diabetes with a high level of certainty (62, 63). Insulin resistance is the pathophysiological mechanism underlying all these predictors (64, 65), while beta cell dysfunction accounts for the progression of pre-diabetes to diabetes and further deterioration of glycaemic control in those with established diabetes (66–68).

This study found a higher prevalence of pre-diabetes and diabetes among women compared to men. This result is similar to the findings of previous studies where more women than men had diabetes (27, 69). The occurrence of major life events has been shown through longitudinal studies to be associated with a rapid increase in HbA1c. For women, this could be attributed to stress, families, and other psychosocial variables (70, 71). Studies have also revealed that increased domestic duties and unpaid labour among women may result in conflicting feelings and possibly stress-induced diabetes (71–73). It could also be attributed to both physiological factors like rising rates of hypertension and obesity in women as well as unfavourable consequences of food and lifestyle choices. Direct causation of diabetes by stressors has not been established. More likely, the stressors unmasked symptoms in people who are already predisposed and possibly in the subclinical stage of the disease process (74, 75). Therefore, it is essential that women receive substantial health education on the risk factors for diabetes in a bid to reduce its prevalence.

## Limitations

This study was carried out in some selected states in the southwest and northcentral zones (only two of the six geopolitical zones in Nigeria); therefore, its findings might not be a representation of other states in Nigeria. In addition, the presence of sickle cell trait among the participants was not excluded, as the potential to cause interference in the HbA1c assay method used in this study could not be completely excluded.

Also, performing both HbA1c and fasting blood glucose simultaneously would have further reduced those that could have been falsely diagnosed as having diabetes or pre-diabetes. However, this approach is more practical and recommended in clinical practice rather than in a population research setting (61). Finally, the cross-sectional nature of the study also did not allow for causal inferences.

Although HbA1c is globally standardised, acceptable, convenient, and useful for screening even in population research, in view of the controversy about the recommended cutoff for dysglycaemia in some races including black Africans, a prospective country-wide study extended all over the country is needed to validate its usefulness in Nigerians and recommend an appropriate reference range for our population.

## Conclusion

The burden of pre-diabetes and diabetes assessed by haemoglobin A1c among community dwellers in the southwestern and northeastern states of Nigeria is very high. Additionally, the proportion of those with undiagnosed diabetes is unacceptably high. Obesity and sedentary living are strong predictors of these abnormal glycaemic states. To promote early detection, screening of all adults for confirmation of their diabetic status should be encouraged to obtain true estimates of individuals with pre-diabetes among the Nigerian population. To achieve this nationwide estimate and achieve completeness of data, statistics on pre-diabetes and diabetes should be captured in the National Demographic Health Survey, and this should be regularly updated. Health education campaigns should be organised especially in community settings to improve knowledge of the risk factors for diabetes to achieve a significant fall in its prevalence. Health decision makers, governmental organisations, non-governmental organisations, and health care professionals in Nigeria must collaborate in this regard to reduce the burden of diabetes.

## Data availability statement

The raw data supporting the conclusions of this article will be made available by the authors, without undue reservation.

## Ethics statement

The studies involving human participants were reviewed and approved by Joint University of Ibadan and University College Hospital Ethical Committee; National Health Research Ethics Committee of Nigeria. The patients/participants provided their written informed consent to participate in this study.

## Author contributions 

IOA contributed to the conceptualisation, design of the study, overall project administration, and data collection, analysis, and interpretation and initiated the writing of the original draft and manuscript review. WB contributed to the conceptualisation and design of the study; assisted in the project administration and data collection, analysis, and interpretation; and contributed to the writing of the original draft, manuscript review, and editing. OBO, GA, DS, OB, MO, and BA-M contributed to the design of the study, data collection, and interpretation, manuscript review, and editing. OO, OF, FD, EO, IA, PA, AA, OA, AO, EG-A, IO, OE, TE, AL, FO, AY, TA, AO, AHA, AB, and SN contributed to the data collection and interpretation, and manuscript review and editing. All authors contributed to the article and approved the submitted version.
